# Building a Social Mandate for Climate Action: Lessons from COVID-19

**DOI:** 10.1007/s10640-020-00446-9

**Published:** 2020-07-08

**Authors:** Candice Howarth, Peter Bryant, Adam Corner, Sam Fankhauser, Andy Gouldson, Lorraine Whitmarsh, Rebecca Willis

**Affiliations:** 1 Grantham Research Institute on Climate Change and the Environment and Centre for Climate Change Economics and Policy, London School of Economics and Political Science, London, UK; 2 Shared Future, Manchester, UK; 3 Climate Outreach, Oxford, UK; 4 Centre for Climate Change Economics and Policy, University of Leeds, Leeds, UK; 5 Centre for Climate Change and Social Transformations, Cardiff University, Cardiff, UK; 6 Lancaster Environment Centre, Lancaster University, Lancaster, UK

**Keywords:** Behaviour change, Climate change, COVID-19, Deliberative governance, Social mandate

## Abstract

The COVID-19 imposed lockdown has led to a number of temporary environmental side effects (reduced global emissions, cleaner air, less noise), that the climate community has aspired to achieve over a number of decades. However, these benefits have been achieved at a massive cost to welfare and the economy. This commentary draws lessons from the COVID-19 crisis for climate change. It discusses whether there are more sustainable ways of achieving these benefits, as part of a more desirable, low carbon resilient future, in a more planned, inclusive and less disruptive way. In order to achieve this, we argue for a clearer social contract between citizens and the state. We discuss how COVID-19 has demonstrated that behaviours can change abruptly, that these changes come at a cost, that we need a ‘social mandate’ to ensure these changes remain in the long-term, and that science plays an important role in informing this process. We suggest that deliberative engagement mechanisms, such as citizens’ assemblies and juries, could be a powerful way to build a social mandate for climate action post-COVID-19. This would enable behaviour changes to become more accepted, embedded and bearable in the long-term and provide the basis for future climate action.

## Introduction

There has been no shortage of commentary on what we can learn from the COVID-19 pandemic for climate change. The pandemic has caused misery, loss and hardship across the world, and should emphatically not be seen as a model for climate action. However, early polling suggests that cleaner air, less traffic, a less frantic pace of life and a less wasteful relationship with food are seen as positive side-effects of the lockdown policies (Pritchard 2020). Thus, there may be opportunities to bed-in and maintain certain types of behaviour changes that would be positive low-carbon steps. There are also tentative signs that the public recognises the need for a response to climate change that mirrors the ambition of our response to the pandemic (Stone 2020).

But there are also serious risks that after having had a ‘taste’ of restrictions on travel and consumption choices, many people will recoil from the idea that (even on a lesser scale, and with careful planning) some of these restrictions or adjustments should continue. With the tangible prospect of a global recession that could dwarf the impact of the global financial crisis of 2008, countries and citizens alike may find it harder to justify investing financially in low-carbon choices, even if the economic logic of making the right low-carbon choices is unquestionably robust (Hepburn et al. 2020).

This commentary explores what responses to COVID-19 can tell us about what may and may not be possible or desirable in a transition to a net-zero future. We have seen how quickly and effectively governments can intervene to completely reshape society and lifestyles; and that society in turn has largely been supportive of this intervention in service of a widely recognized urgent health threat. This level of intervention however has rarely been observed previously, except during war-time. But while it may be possible for governments to do this, is it possible or desirable for them to do so in order to address climate change?

We argue that a rapid zero-carbon transition is possible, and that with the right policies some of the behaviour changes that the lockdown has imposed might be sustained. However, such a transformation needs to be underpinned by a clear social mandate and public support, and it needs to be well planned to avoid the disruptive effects of COVID-19. By a social mandate, we refer to a situation where society offers support to another actor (e.g. government) to take action to protect our collective well-being, with the processes and the outcomes of this action being broadly accepted as being legitimate. Such a mandate is also sometimes referred to as a social contract or a social license for action. Deliberative citizen engagement can support both these objectives.

## Two Different Kinds of Emergencies

The two problems of COVID-19 and climate change are strikingly similar and different at the same time. Both have been described as global emergencies. Both raise the prospect (indeed the reality) of unprecedented risks to human health and prosperity. Both problems are global, need individuals to act for the common good, require government intervention in lifestyles, challenge societal resilience, and have some common solutions (e.g., travelling less).

COVID-19 has increased our awareness of how vulnerable we can be in the face of global risks, whether a pandemic or climate change, and how without foresight and planning we are left ill-prepared. The two problems share common drivers—including global travel, deforestation and land-use change—and are mutually reinforcing—climate change is known to affect the survival, reproduction, abundance, and distribution of pathogens, vectors, and hosts (Wu et al. 2016).

However, the two emergencies are also different in important ways. While the threat of COVID-19 is immediate and direct, the impacts of climate change are longer term and more diffuse. Driven by necessity, the global response to COVID-19 has been radical and swift. The global response to climate change, in contrast, has lacked a sense of urgency (despite a large number of climate emergencies being declared).

Perhaps this is because climate change is less tangible and emotive than COVID-19: the victims of climate change are less likely to be people we know (or ourselves), and even how we measure climate change (in e.g., greenhouse gas emissions or temperature increase) is more abstract and less visceral than how we measure COVID-19 (in number of deaths). Indeed, health concerns usually rank higher than environmental ones, because they are more psychologically proximal (Maibach et al. 2010). There is lower self-efficacy in mitigating climate change than COVID-19: the latter risks to the individual can be mitigated by hand-washing and social distancing, whereas no amount of individual energy saving will necessarily mitigate climate change risks to the individual.

There is a lower social norm to act on climate change than on COVID-19: simply looking out of the window during lockdown, we can see others are (largely) complying; but low-carbon lifestyles are a long way off being ‘normal’ or even aspirational. This social norm is, of course, partly a product of stricter government regulation in the case of COVID-19 than climate change. The timescale for interventions is also different: lockdown and other COVID-19 responses are likely to be relatively short-term (months or perhaps years); whereas climate change policies may be in place indefinitely.

## Making New Behaviour Last

We have learnt how quickly people can adapt their lives to a sudden disruption like a pandemic-induced lockdown. The social response to this exogenously enforced shock is likely to have varied in different countries reflecting the social norms and contracts in place in those different contexts. For example the use of draconian restrictions on movement such as fines and permits in France and Italy (and the rapid behaviour change resulting from this) reflects the culture, role and acceptance of law enforcement within those countries; an approach less accepted in more libertarian countries such as the UK. Typically, one of the biggest barriers to behaviour change are habits, and we know times of disruption are when we can most effectively change habits (Verplanken et al. 2019; Graham-Rowe et al. 2011). COVID-19 is the biggest moment of disruption we have seen since World War 2 and, while a tragedy, it has created some behavioural patterns that are intrinsically low-carbon (less travel, less consumption in some areas). They could have great value in shaping the response to climate change, but it is not yet clear how durable they will be.

It takes on average 66 days to create a new habit. So, after 10 weeks, new patterns of behaviour may become routines that stick with us (Lally et al. 2010). In most countries, the lockdown has lasted at least 10 weeks, suggesting new ways of living might endure post-lockdown. However, this is highly contingent on these habits being locked in by appropriate infrastructure, incentives and norms (e.g., continuing to allow people to work from home at least some days; providing more cycling infrastructure) and ideally aligned with people’s core values (e.g., spending time with family; environmental protection; Verplanken et al. 2019). Together, this ensures the motivation and ability to maintain low-carbon habits.

The extent to which policies on COVID-19 are driven by ‘consent’, as opposed to ‘necessity’ also needs consideration. COVID-19 has shown that, under certain conditions, the relationship between citizen and state is far more elastic than previously thought. Citizens have relied upon the state to keep them safe, imposing measures that would have previously been dismissed as unthinkable. For their part, citizens have accepted and encouraged these measures, with compliance levels beyond what was expected or modeled, as suggested by evidence from the UK. The modelling of non-pharmaceutical interventions by Imperial College (Ferguson et al. 2020), used by the UK government to plan lockdown measures, assumed compliance rates of 50–75%, whereas surveys in April 2020 showed over 80% compliance with social distancing (Weinberg 2020).

However, the lockdown measures were—until days before being in place—unforeseen and implemented with lead times considerably shorter compared to normal democratic processes (which would include consultations, white papers, debates in parliament), considering the speed at which the virus was spreading (e.g. arriving in Italy in February and the UK in March). There was therefore very little scope for undergoing a weighing up of values, trade-offs and competing priorities that any other far-reaching policy issue would require as standard democratic practice (Hagendijk and Irwin 2006). The emergency measures brought in to freeze almost all ‘in person’ social and economic activity were driven by necessity, rather than based on considered societal choices. While unplanned transitions like the COVID-19 response are thus workable in the short-term, they may be difficult to maintain in the long-term.

## Minimising Disruption

While COVID-19 has proven that radical behavioural change is possible, in the short-term, the economic, social and personal consequences of the lockdown have been devastating. The impact on greenhouse gas emissions has been large by historical standards, but modest relative to the deep emission cuts that are needed (Le Quéré et al. 2020). The sudden and disruptive response to COVID-19 is in stark contrast to the sustained, carefully calibrated but urgent and long-term response that climate change requires.

The more deliberate planning of the zero-carbon transition is important for two reasons. First, a planned transition will allow people to be engaged and included. Involving publics and stakeholders in climate change decision-making not only tends to improve the quality of decision-making (by incorporating a broader range of views and expertise, Howarth et al. 2017), it also builds trust in the process and buy-in to the outcomes (Dietz and Stern 2008).

Second, planning a transition provides more time to minimise negative impacts and to maximise the co-benefits associated with tackling climate change. While the hurried nature of the COVID-19 response was inevitably costly, the evidence is growing that a well-executed zero-carbon transition can be achieved without compromising on economic prosperity (Kruse et al 2020; Bowen and Hepburn 2014). A carefully planned response allows identification of those areas where the structural adjustment costs of decarbonization are real and putting in place safeguards for a just transition (Bowen and Fankhauser 2017; Newell and Mulvaney 2013). Planning will allow exploitation of the co-benefits associated with mitigating climate change, notably for health (e.g., reducing car use improves air quality and reduces obesity, limited red meat consumption cuts heart disease and cancer risks; Jennings et al. 2019).

The complexity of these measures, together with genuine uncertainty and disagreement about how to address climate change in an effective and fair way (Hulme 2009), means that climate change tends to be a less salient concern for individuals, organisations and policy-makers than more tangible and immediate issues, not least COVID-19 at the moment—but more generally, personal and family health, financial security, and social/community cohesion (e.g., Bain et al. 2016; Whitmarsh and Corner 2017).

This means that measures that align emission reductions with those individual concerns are more likely to motivate behaviour and policy change (Graham and White 2016; Whitmarsh 2009). But it also speaks to the imperative of securing a strong social mandate for continuous change beyond COVID-style emergency measures.

## Guided by the Science

COVID-19 has highlighted the role of experts in informing government responses to the pandemic crisis. Scientists have been called upon to inform and influence on an issue fraught with uncertainties (Lövbrand and Öberg 2005). The prominence of government committees like the Scientific Advisory Group for Emergencies (SAGE) in the UK has prompted widespread debate about the role of science in policymaking. When faced with an issue like COVID-19, politicians often claim that their decisions are based on ‘the science’, or that ‘science’ itself is somehow making the decisions. This is a problematic presentation for two reasons. First, because the science itself is not value-free, and neither are the scientists who promote it. Second, because scientific evidence cannot, in and of itself, determine political decisions—it can only inform them. Political decisions are moral and ethical choices in conditions of uncertainty, not merely a product of machine-like evidence-processing (Wilsdon and Willis 2004).

Thus the role of political decision-making should be seen as a negotiation between different actors, including scientists, policymakers, interest groups (such as businesses, NGOs, professional associations and trade unions) publics and others, informed by scientific and social scientific evidence (Davies and Oreszczyn 2012). Involving diverse groups in decision-making processes—and crucially, including those who policies will impact—leads to more robust outcomes (Stirling 2008). This is as true for COVID-19 as it is for climate action. The rapid onset of the pandemic in the first half of 2020 meant that decisions were taken very quickly, which did not allow widespread consultation and engagement. People accepted lockdown measures because they understood the benefits, in terms of saving lives (including their own), and because the most restrictive measures were seen as relatively short-term. As countries begin to lift strict lockdowns, learn to live with the virus, and begin the process of economic and social recovery, it is both possible and necessary to widen the decision-making process (Smith and Hughes 2020). Crucially, this should include deliberative democratic processes such as citizens’ juries, which we discuss below.

For the reasons discussed above, support for government intervention to address climate change is less visible and more uncertain than for COVID-19. Scientific understanding of climate change is very well established, has been built up over many years, and there is a very high degree of consensus; there is also good understanding of the measures required to bring emissions down to safe levels (IPCC 2018). However, there is limited debate, and no societal consensus on *how* we reach net zero, in terms of which measures governments should implement. There is public support for climate change action, including a low-carbon economic stimulus post-lockdown (Figueres 2020), but there is a need for a more comprehensive debate, bringing scientific evidence together with public views and values—a key characteristic of deliberative governance activities such as citizen juries and assemblies (Kythreotis et al. 2019).

## Building a Social Mandate for a Change

In considering climate policy, politicians have been reluctant historically to contemplate imposing restrictions upon citizens. They did not feel under pressure from the electorate to act on climate (Willis 2017) and prioritised policies which they perceived were ‘neutral’ in terms of lifestyle—such as switching to grid-based renewables or shifting from petrol and diesel to electric vehicles. The school strikes and climate protests which began following the publication of the 2018 report of the Intergovernmental Panel on Climate Change (IPCC 2018) have helped to persuade politicians of the importance of this agenda, but they are still reluctant to speak out about the fundamental changes that are required to shift economies and societies to net-zero emissions (Willis 2019).

The pandemic has not necessarily changed the political calculus. The fact that drastic measures were accepted for COVID-19 does not imply they would be accepted for climate. The lesson from COVID-19 is more subtle: it shows that the challenge for climate strategy is not to assume, as politicians have done, a limited room for manoeuvre on climate, but rather to work with citizens to explore what is possible. Using the language of political theory, this can be thought of as the negotiation of a contract between state and citizen to build a social mandate for change. Such a mandate consists of contributions from both sides—state and citizen—each conditional upon the actions of the other side (Boucher and Kelly 2003).

This is where deliberation comes in. Traditional polling measures and social research, can explore people’s attitudes and values on particular issues, such as their views on renewable electricity technologies. But the fundamental question about the social mandate requires deliberation—an exchange between citizens and state (Dryzek 2002). Citizen juries and assemblies are a tool out of several, which can help to create the social mandate to move forward on socially-inclusive climate action. By co-producing and including citizen’s input into designing solutions they help increase public trust and ensure publics are on board and more receptive to any conditions (behavioural or other) that are implemented (Warren and Gastil 2015).

COVID-19 interrupted an extraordinary wave of grassroots climate activism and experimentation with deliberative forms of climate governance. In the UK, for example, two thirds of local councils declared a climate emergency in 2019, and many complemented their emergency declarations with the establishment of a citizens’ assembly or citizens’ jury. A national-level Climate Assembly sat between January and May 2020 in the UK (Climate Assembly UK 2020) and, similarly, the French Citizen’s Convention met seven times from October 2019 to June 2020 (Convention Citoyenne pour le Climat 2019), the impacts of which will need to be analysed.

Some of these citizen’s assemblies and juries are already having an impact (e.g. in Leeds, UK, the process led to substantial citizen engagement and recommendations on what the city should do to address the climate emergency), however it is too early to tell what the widespread impact of citizens assemblies (e.g. in France and the national UK assembly) on the climate debate has been. Citizen assemblies have certainly been influential in other contexts, such as the Irish debate on abortion (Farrell et al. 2019). To its supporters, they have demonstrated the power of randomly selected citizens that reflect the wider population, coming together to navigate their way through complexity to create a set of citizen-led visions and recommendations for the future.

As such, these ‘mini publics’ can be an important instrument to obtain a social mandate for the zero-carbon transformation. They are a structured way of equipping citizens with a coherent and robust narrative on climate change, supporting citizens to imagine different ways of living and giving politicians the mandate to take action. They make society a co-designer of climate action rather than having solutions imposed on them (Capstick et al. 2020).

Deliberative processes including Citizens’ Assemblies and Citizens’ Juries have involved in-person meetings, which is problematic given the restrictions that COVID-19 imposes. But innovation is happening quickly: the UK’s Climate Assembly moved online in April 2020, and initial evaluation by participants showed that it worked remarkably well (Allan 2020). The first entirely-online Citizens’ Jury on climate change is about to start, in the town of Kendal. Such processes can test to what extent people have emerged from the pandemic with different values, altered behaviours and perhaps an increased appetite to envision and shape their own future. Citizen assemblies may not always generate the results that we expect, but by encouraging collective debate and decision-making they frequently propose a more positive future and different ways of getting there, which people are ready to support.

## Conclusions

COVID-19 has increased our awareness of how vulnerable we can be in the face of global phenomena, and how without foresight and planning we are left ill-prepared. We have been made aware of our co-dependence and how we are citizens in a society as well as individuals in an economy. The pandemic has strengthened the case for an economic recovery that puts emissions reduction, and indeed climate resilience, at its heart.

The global response to COVID-19 has had environmental side effects that the climate community has aspired to achieve over a number of decades: reduced carbon emissions, cleaner air, less noise, more space for nature. However, these benefits have been achieved at a massive cost to welfare and the economy. The COVID-19 response is therefore not a suitable model for climate action. Climate change requires a more carefully planned and calibrated, inclusive, less disruptive and more sustained response.

We argue that in order to accomplish such a response, there is a need for a clearer social mandate between citizens and the state. We suggest that deliberative engagement mechanisms, such as citizens’ assemblies and juries, could be a powerful way to build a social mandate for climate action post-COVID. This would enable behavioural changes that improve wellbeing and underpin climate action over the years ahead.
